# Fat Amount Rather Than Fatty Acid Composition Influences Postprandial Hunger, Satiety and Attention in Men and Women with a Risk Phenotype for Cardiometabolic Diseases: A Randomized Crossover Trial

**DOI:** 10.1016/j.tjnut.2025.11.003

**Published:** 2025-11-11

**Authors:** Christina Diekmann, Tim B Schiemann, Hannah F Kienēs, Carolin Wiechmann, Christina Kopp, Birgit Stoffel-Wagner, Martin Coenen, Robert Németh, Michael Wagner, Sarah Egert

**Affiliations:** 1 Institute of Nutritional and Food Sciences, Nutritional Physiology, University of Bonn, Bonn, Germany; 2 Institute of Clinical Chemistry and Clinical Pharmacology, University Hospital Bonn, Bonn, Germany; 3 Institute of Medical Biometry, Informatics and Epidemiology, University Hospital Bonn, Bonn, Germany; 4 Department of Old Age Psychiatry and Cognitive Disorders, University Hospital Bonn, Bonn, Germany; 5 German Center for Neurodegenerative Diseases (DZNE), Bonn, Germany

**Keywords:** postprandial metabolism, canola oil, coconut oil, satiety, attention

## Abstract

**Background:**

Hunger and satiety are influenced by both the amount and composition of dietary fat.

**Objectives:**

This exploratory analysis examined the effects of meals enriched with different amounts of either canola oil (rich in unsaturated fatty acids) or coconut oil (rich in saturated fatty acids) on postprandial changes in subjective hunger and satiety ratings, related hormones, attention, and memory.

**Methods:**

Twenty-nine participants with an increased cardiometabolic disease risk (e.g., body mass index, 30.2 ± 2.6 kg/m^2^) received 4 isoenergetic (∼4200 kJ) test meals in a randomized order: high-fat meals (HFMs) (50 g) or low-fat meals (LFMs) (25 g) containing either canola or coconut oil. Hunger and satiety ratings, blood samples for ghrelin and peptide YY (PYY), and cognitive tests were conducted at fasting and over a 6-h postprandial period. The data were analyzed via linear mixed models.

**Results:**

Lower serum ghrelin levels were observed after canola oil meals [canola compared with coconut: –27,700 (confidence interval: –40,700, –14,700) min × pg/mL] with no effect of fat amount on incremental area under the curve data [HFM compared with LFM: –9500 (–22,500, 3500) min × pg/mL]. Serum PYY concentrations were higher after HFMs [HFM compared with LFM: 8600 (2100, 15,200) min × pg/mL]. LFMs resulted in lower subjective hunger ratings compared with HFMs [HFM compared with LFM: 250 (71, 430) min × score-value].

**Conclusions:**

Our data indicate that predominantly the amount of dietary fat, rather than its type, affects hunger and satiety. At the hormonal level, canola oil suppressed postprandial hunger to a greater extent than coconut oil.

This trial was registered at ClinicalTrials.gov (https://clinicaltrials.gov/) as NCT05208346.

## Introduction

Eating behavior is regulated by a complex interplay of homeostatic and nonhomeostatic mechanisms [1]. In this context, hedonic/reward-related pathways, which are involved in the pleasurable perception of food, can override energy balance signals, driving food intake beyond physiological needs. This dysregulation can contribute to excessive energy intake and thus plays a critical role in the development and progression of obesity [2,3]. Gastrointestinal signals are crucial in the homeostatic regulation of hunger and satiety. In this context, not only the gastric distension and emptying time are relevant, but also the interaction of nutrients with receptors in the small intestinal mucosa, which contributes to the release of anorexigenic [e.g., peptide YY (PYY)] and orexigenic (e.g., ghrelin) gut peptides and neurotransmitters [4]. Studies have shown extensively that macronutrients exert differential effects on satiety, with protein considered to be the most satiating macronutrient per kilojoule, followed by carbohydrates and fat [5]. The satiating effects of dietary fat are mediated through multiple mechanisms, including the stimulation of anorexigenic peptide secretion, as well as the inhibition of gastric emptying and intestinal transit [6]. To date, only a few human intervention studies have investigated whether, in addition to the total amount of fat, the fatty acid composition (i.e., chain length and degree of saturation) influences hunger and satiety and the results remain inconclusive [[6], [7], [8], [9], [10], [11], [12], [13], [14], [15], [16], [17], [18]]. Regarding satiety-related hormones, research data suggest that the PYY response increases according to longer fatty acid chain length [11,19]. Furthermore, the degree of saturation seems to influence PYY secretion by affecting the duration of absorption and, consequently, the contact time of fatty acids with PYY-secreting L-cells in the small intestine [15,19]. Animal studies further indicate that high-fat meals differentially affect the expression of hypothalamic orexigenic and anorexigenic neuropeptides, depending on the fatty acid composition; however, this mechanism has not yet been investigated in human studies [7].

To the best of our knowledge, the acute effects of canola and coconut oil on hunger, satiety, and cognitive parameters in a clinically relevant cohort characterized by an increased risk of cardiometabolic diseases (e.g., older age; characteristics of metabolic syndrome) have not been investigated thus far. Both oils differ in their fatty acid composition. Canola oil is rich in unsaturated fatty acids (MUFA and PUFA, ∼89% of total fatty acids) and coconut oil is rich in SFAs (∼94% of total fatty acids) (Table 1) [20]. In addition, canola oil primarily contains longer-chain fatty acids, whereas coconut oil also includes medium-chain fatty acids in smaller amounts (Table 1) [20]. By designing 4 different test meals with varying amounts of canola or coconut oil, we were able to investigate the effect of fat type/fatty acid composition, as well as of fat amount, on the defined outcome measures. The presented data were obtained as secondary outcome measures in a trial originally designed to investigate the postprandial effects of canola and coconut oils on metabolic, inflammatory, oxidative, and endothelial parameters [20]. Therefore, the data presented here represent ancillary examinations.

**TABLE 1:** Fatty acid composition of the test oils (adapted from Kienēs et al. [20]).

Component	Canola oil	Coconut oil
Fatty acids	% of total fatty acids	
Caprylic acid (8:0)	nd	4.68
Capric acid (10:0)	0.01	5.82
Lauric acid (12:0)	0.01	52.53
Myristic acid (14:0)	0.05	20.06
Palmitic acid (16:0)	4.38	8.42
Stearic acid (18:0)	1.46	3.14
Oleic acid (18:1n-9)	62.27	4.36
Linoleic acid (18:2n-6)	19.15	0.71
α-Linolenic acid (18:3n-3)	7.24	nd

Abbreviation: nd, not detectable (< 0.01 % of total fatty acids).

On the basis of the current research regarding the impact of fat amount and fatty acid composition on satiety-related peptides, we focused on 2 primary research questions: *1*) Do meals containing canola oil elicit a stronger increase in PYY compared with meals containing coconut oil (fat type effect)? and *2*) Do high-fat meals result in a greater increase in PYY compared with lower-fat meals (fat amount effect)? Fat ingestion has been shown to suppress the ghrelin response less effectively than carbohydrate or protein intake, and the potential influence of different fatty acids (SFAs, MUFAs, PUFAs) on the postprandial ghrelin response has only been scarcely investigated [21,22]. Therefore, we examined the effects of both fat amount and fatty acid composition on postprandial serum ghrelin. In addition, we investigated the effects of fat amount and fatty acid composition on subjective hunger and satiety ratings, as well as postprandial attention and memory.

## Methods

### Participants

The inclusion criteria for study participation were as follows: *1*) age between 60 and 80 y, *2*) overweight or obesity (BMI 27–34.9 kg/m^2^), and *3*) visceral fat distribution (waist circumference ≥94 cm for men and ≥80 cm for women). Furthermore, ≥2 of the following characteristics of metabolic syndrome had to apply [23]: *1*) dyslipidemia (serum triglycerides ≥1.7 mmol/L and/or serum HDL cholesterol <1.03 mmol/L for men and <1.29 mmol/L for women), *2*) increased resting blood pressure (systolic BP ≥130 mmHg and/or diastolic BP ≥85 mmHg), and *3*) increased plasma glucose (≥5.6 mmol/L). Exclusion criteria comprised smoking, malabsorption syndromes, untreated thyroid diseases, impaired kidney function, myocardial failure, insulin-treated diabetes mellitus, chronic inflammatory diseases, cancer, alcohol or drug abuse, epilepsy, anemia, immunosuppression, long-term intake of certain supplements (especially marine n-3 fatty acids and vitamin E), and participation in another intervention study within the last 30 d.

Interested volunteers (*n* = 86) attended a screening that included physical assessments (body height and weight, waist and hip circumference, fat and fat-free mass, resting BP, heart rate), clinical assessment (parameters of liver and kidney function, serum lipids and lipoproteins, plasma glucose, serum C-reactive protein, blood count), medical history, and the documentation of dietary habits. In total, 30 participants (18 men, 12 women) were included in the study. During the intervention period, 1 woman dropped out because of difficulties in fulfilling the requirements of the study protocol. Because of the absence of detectable measurements for ghrelin and PYY in 3 participants, these individuals were excluded from the statistical analysis. Consequently, the analysis was conducted exclusively on complete datasets (*n* = 26 for ghrelin and PYY, *n* = 29 for all other outcome measures).

Written informed consent was obtained from all participants prior to inclusion. The study was conducted in accordance with the guidelines laid down in the 1964 Declaration of Helsinki and its later amendments. All study procedures were approved by the ethics committee of the Medical Faculty of the University of Bonn (ethic approval code 420/21). The study was registered at clinicaltrials.gov (https://clinicaltrials.gov/) as NCT05208346.

### Study design

The postprandial study was carried out at the Institute of Nutritional and Food Sciences, Nutritional Physiology (University of Bonn). It was conducted in a randomized crossover design and comprised 4 treatment conditions, each lasting 6 h from morning until afternoon: *1*) 50 g canola oil–containing meal (“canola oil high-fat meal”), *2*) 25 g canola oil–containing meal (“canola oil low-fat meal”), *3*) 50 g coconut oil–containing meal (“coconut oil high-fat meal”), and *4*) 25 g coconut oil–containing meal (“coconut oil low-fat meal”). The order of treatments was randomized prior to commencement of the study for each participant via 4 × 4 Williams design by the cooperating biometrician (RN) using a statistical software (SAS version 9.4). Participants were assigned to their sequence of interventions by study personnel prior to the commencement of the study. The 4 treatment days were separated by ∼2 wk wash-out periods.

On each treatment day, venous blood sampling for ghrelin and PYY analysis were taken at fasting (0 h) and at various time points during the 6-h postprandial period (1.0, 2.0, 4.0, and 6.0 h). Questionnaires for attention, hunger, and satiety were carried out at fasting (0 h), at 1.5, 3.0, 4.5 (attention), and 0.5, 1.0, 2.0, 4,0, 6.0 h (hunger and satiety) in the postprandial state. The questionnaire for memory was performed directly after meal ingestion (0.5 h postprandially) as well as at 1.5 and 6.0 h postprandially. Blood drawing and analyses as well as statistical analysis occurred in a blinded manner (study personnel).

### Test meals

The 4 test meals were specifically designed for the purpose of this study. The meals were isoenergetic (∼4200 kJ/meal) and iso-nitrogenous (26.6 g protein/meal). Each meal contained 50 g (high-fat meals) or 25 g (low-fat meals) canola oil (Brölio canola oil; Brökelmann + Co) or coconut oil (virgin cold-pressed coconut oil; Schneekoppe) and therefore differed in fat amount and fatty acid composition. Furthermore, the carbohydrate content, as well as the amount of dietary fiber, were higher in the low-fat meals than in the high-fat meals (Table 2) [20]. The main components of the test meals were a homemade vegetable soup and other commercially available foods such as baguette, yogurt, jam, and fruit juice. The test oils were mixed into the yogurt, as this provided the most homogeneous distribution.

**TABLE 2:** Energy content and nutrient composition per serving of the test meals (adapted from (23))

	Canola oil HFM	Coconut oil HFM	Canola oil LFM	Coconut oil LFM
Energy (kJ)	4187	4202	4192	4200
Carbohydrates (g)	85.5	85.5	144.1	144.1
Carbohydrates (EN%)	35	35	59	59
Dietary fiber (g)	6.6	6.6	11.2	11.2
Protein (g)	26.6	26.6	26.6	26.6
Protein (EN%)	11	11	11	11
Total fat (g)	60.9	60.5	33.6	33.4
Total fat (EN%)	54	54	30	30
SFAs (g)	8.2	45.7	5.9	24.6
Lauric acid (12:0) (g)	0.2	21.1	0.2	10.7
Myristic acid (14:0) (g)	0.5	8.8	0.6	4.8
Palmitic acid (16:0) (g)	4.6	6.8	3.0	4.0
SFAs (EN%)	7.3	40.2	5.2	21.7
MUFAs (g)	35.5	7.0	18.4	4.2
Oleic acid (18:1n-9) (g)	34.2	6.5	17.6	3.8
MUFAs (EN%)	31.4	6.2	16.2	3.7
PUFAs (g)	15.8	2.5	8.4	1.8
Linoleic acid (18:2n-6) (g)	10.9	2.2	5.8	1.4
α-Linolenic acid (18:3n-3) (g)	4.8	0.3	2.6	0.3
PUFAs (EN%)	14.0	2.2	7.4	1.6
Cholesterol (mg)	210	210	15	15
Vitamin E^1^ (mg)	14.5	2.5	8.2	2.2
Vitamin C (mg)	43.6	43.6	60	60

Abbreviations: EN%, energy percentage; HFM, high-fat meal; LFM, low-fat meal.

1 α-tocopherol equivalents.

All test meals were prepared by study personnel at the study center each study day morning, according to a standardized protocol involving the weighing of every food item to the exact gram. Participants consumed the test meals as their first meal of the day (breakfast challenge) in the morning after a 10-h overnight fast. The test meals were required to be consumed within 20 min under supervision of study personnel.

### Measurements

#### Fasting and postprandial parameters in serum (ghrelin and PYY)

Details of the preanalytical procedures of fasting and postprandial blood samples have previously been described [20]. Fasting and postprandial (1.0, 2.0, 4.0, and 6.0 h) ghrelin (total) and PYY (total) were analyzed in duplicate from –80°C frozen serum samples using commercially available enzyme-linked immunoassay kits (Merck Millipore KGaA) according to the manufacturer’s instructions and recommended quality control procedures.

#### Hunger and satiety ratings

On all study visits, the sensations hunger and satiety were assessed using 100-mm visual analog scales (VAS), which are a valid, reliable, and objective measuring instrument with high sensitivity to changes [24,25]. On the varying VAS, the sensations were paired with the correspondingly opposite sensations (“not hungry”/“hungry,” “not satiated”/“satiated”). In the fasting state (0 h) and at 0.5, 1.0, 2.0, 4.0, and 6.0 h in the postprandial state, participants were requested to make a vertical mark that best matched their current feeling on each scale. For evaluation purposes, each score was determined by measuring the distance from the left side of the line to the vertical mark.

#### Cognitive parameters

Visual selective attention as 1 facet of cognitive function was measured using the validated paper pencil test Frankfurt Attention Inventory 2 (FAIR-2, German version) [26,27]. Both test versions (A and B) were used in an alternating, randomized order at each study day. The test versions each comprise 2 x 320 items (target items and nontarget items) that are arranged in rows. To complete the test, the target items must be identified and marked among the similar nontarget items by drawing a continuous line under each row of items with a “spike” extending from it, clearly marking each target item. Both accuracy and speed are combined into an overall performance score (K score) to evaluate test performance. The FAIR-2 was conducted in the fasting state (baseline) and 1.5, 3.0, and 4.5 h postprandially on each of the 4 study days in a distraction-free environment under the guidance and supervision of study personnel.

Memory was tested using the Visual and Verbal Memory Test (Visueller und Verbaler Merkfähigkeitstest, VVM) [28]. This validated test procedure exists in 4 equivalent versions, all of which measure the retention of memorized information. Each of the 4 VVM-versions comprises a test form for visual memory (visual test form) and verbal memory (verbal test form). In the visual test form, a marked route from A to B, shown on a map, is memorized by the participants (learning phase) and must then be transferred to a blank city map (recall/reproduction phase). In the verbal test form, the participants are presented with information about a building which they have to memorize (learning phase) and later are queried with the help of a questionnaire (recall/reproduction phase). In our study the participants were initially confronted with the VVM at 0.5 h postprandially (learning phase and subsequent recall/reproduction phase). The recall/reproduction phase was then repeated at 1.5 h and 6.0 h in the postprandial state. At each of the 4 study visits, 1 of the 4 VVM-versions was used in a predefined order. All tests were conducted in a distraction-free environment under the guidance and supervision of study personnel. The test evaluation included the calculation of forgetting rates between the individual query times (short-term forgetting: 0.5–1.5 h postprandial; longer-term forgetting: 0.5–6.0 h postprandial) on all 4 study days for both the visual and verbal test forms.

### Statistics

The presented data were obtained as secondary outcome measures in a trial originally designed to investigate the postprandial effects of canola and coconut oils on metabolic, inflammatory, oxidative, and endothelial parameters [20]. Therefore, the data presented here represent ancillary examinations and were not considered during the original sample size calculation.

All statistical analyses were performed using the IBM SPSS statistical software package (SPSS version 29, IBM Corporation). Data were analyzed according to a prespecified statistical analysis plan, which was finalized in cooperation with the collaborating biometrician (RN) before outcome data were available. The linear mixed model procedure with repeated measures was used to test the effects of fat amount (high-fat, low-fat), fat type (canola oil, coconut oil), postprandial time points (VAS: 0.5, 1.0, 2.0, 4.0, and 6.0 h; FAIR-2: 1,5, 3.0, and 4.5 h; ghrelin and PYY: 1.0, 2.0, 4.0, and 6.0 h), and their interactions on subjective hunger and satiety ratings (VAS data), attention (K score, FAIR-2), serum ghrelin and serum PYY. The factors fat amount, fat type, postprandial time points, and their interactions were set as fixed factors. The study visit (period) was also set as fixed factor, but without interaction with the other fixed factors. The subject identifier was set as a random factor and the baseline (fasting) value of the specific outcome measure was included as a covariate in the respective model (if applicable), to adjust for baseline differences. In all tests, the residuals were checked for normal distribution and the linear mixed model was rerun with log-transformed data in case of clear violation of the normality assumption (PYY). In addition, the incremental AUC (iAUC or net iAUC) was calculated for all time-dependent parameters using the linear trapezoidal rule. A linear mixed model was fitted to evaluate the effect of fat amount, fat type, and their interaction, while also accounting for the design effects (study visit/period and subject effect). Such iAUC values represent a weighted average over time and it is expected that potentially observed differences are more robust than in a timewise comparison. Pairwise comparisons were mainly reported for high-fat compared with low-fat and canola compared with coconut. Similar analyses were performed for the VVM forgetting rates, separately for short-term and longer-term forgetting (short-term forgetting: 0.5–1.5 h postprandial; longer-term forgetting: 0.5–6.0 h postprandial). In all analyses, the significance level was set at *P* < 0.05 but should be interpreted in an exploratory manner as the trial was not powered for the objectives of this investigation. All data are presented as arithmetic mean ± SEM, unless otherwise stated. Pairwise comparisons are generally presented as mean differences along with the 95% confidence intervals.

## Results

### Baseline characteristics

The characteristics of the participants at baseline are presented in Table 3 [20]. All participants were overweight (48.3%) or obese (51.7%), had a visceral fat distribution, an increased resting BP, and an increased plasma glucose concentration [20].

**TABLE 3:** Baseline characteristics of study participants in total and per randomization sequence (4 × 4 Williams Design) (adapted from Kienēs et al. [20]).^1^

	Total (*n* = 29)	Sequence 1 (*n* = 8)	Sequence 2 (*n* = 7)	Sequence 3 (*n* = 7)	Sequence 4 (*n* = 7)
Age (y)	70.0 ± 5.3	70.6 ± 5.4	70.6 ± 6.0	68.0 ± 5.8	70.9 ± 4.3
Sex (m/f)	18/11	5/3	4/3	6/1	3/4
BMI (kg/m^2^)	30.2 ± 2.6	30.2 ± 3.2	30.3 ± 2.3	30.6 ± 2.5	29.9 ± 2.9
Fat mass (%)	39.2 ± 8.3	39.3 ± 5.3	41.0 ± 10.7	35.2 ± 8.1	41.4 ± 9.0
Waist circumference (cm)	107.1 ± 7.9	107.3 ± 10.7	104.0 ± 6.3	112.3 ± 6.3	105.0 ± 5.3
Systolic blood pressure (mmHg)	150.7 ± 18.3	149.9 ± 20.5	153.0 ± 13.1	151.6 ± 9.3	148.6 ± 28.2
Diastolic blood pressure (mmHg)	87.4 ± 9.7	88.1 ± 7.9	88.3 ± 9.1	87.1 ± 10.5	86.0 ± 12.9
Serum triglycerides (mmol/L)	1.83 ± 0.77	1.93 ± 1.03	1.67 ± 0.75	2.03 ± 0.78	1.66 ± 0.48
Serum total cholesterol (mmol/L)	5.58 ± 1.22	5.80 ± 0.72	6.42 ± 1.07	5.20 ± 1.30	4.87 ± 1.36
Serum HDL cholesterol (mmol/L)	1.45 ± 0.47	1.24 ± 0.23	1.99 ± 0.48	1.22 ± 0.27	1.40 ± 0.44
Serum LDL cholesterol (mmol/L)	3.51 ± 1.02	3.85 ± 0.61	3.94 ± 0.96	3.19 ± 1.18	3.00 ± 1.16
Plasma glucose (mmol/L)	5.88 ± 1.05	5.59 ± 0.83	5.66 ± 0.28	6.42 ± 1.78	5.91 ± 0.78
Serum hs-CRP (mg/L)	2.05 ± 1.52	2.11 ± 1.55	2.08 ± 2.04	2.27 ± 1.16	1.74 ± 1.46

Abbreviations: f, female; hs-CRP, high-sensitive C-reactive protein; m, male.

1 Baseline characteristics were assessed once at screening and not separately per period. Data shown as mean ± SD.

### Fasting and postprandial parameters in serum (ghrelin and PYY)

**FIGURE 1:**
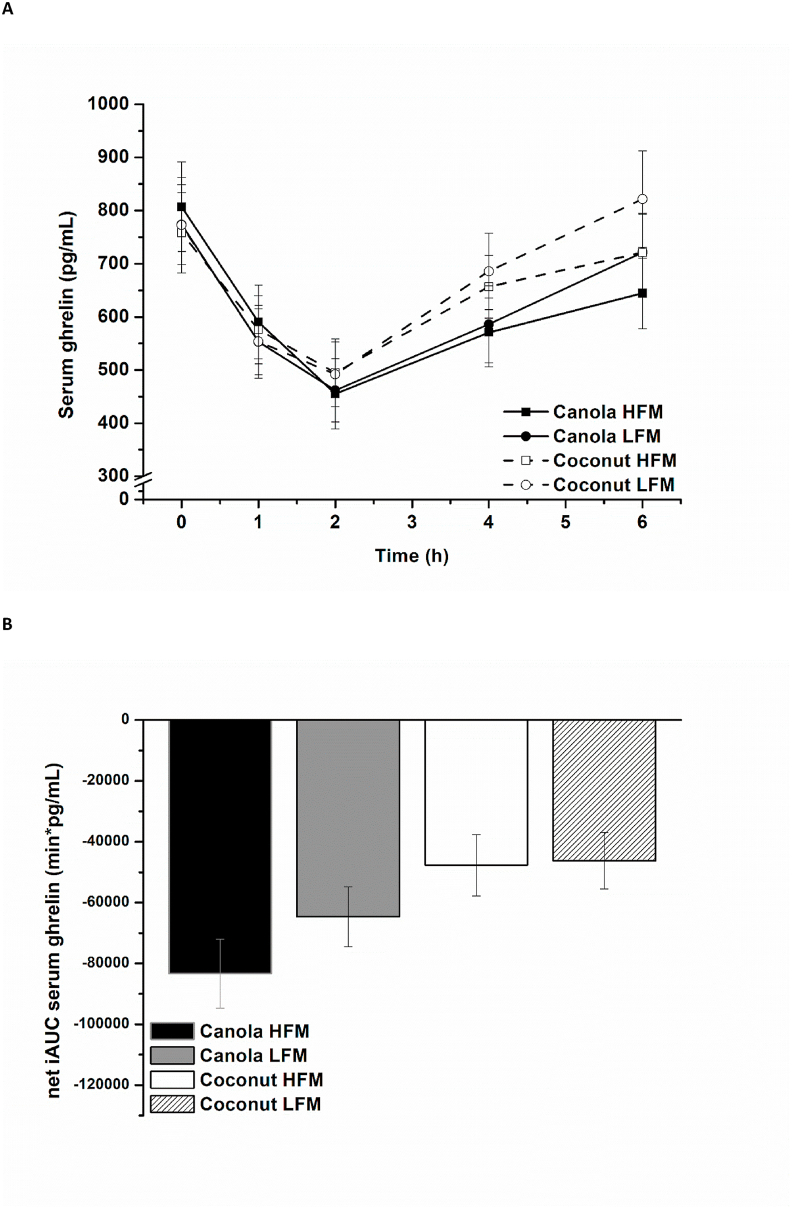
Fasting and postprandial serum ghrelin according to treatment condition shown as mean ± SEM (*n* = 26). (A) Serum ghrelin over time, *P* < 0.001 for fixed factor time and fixed factor fat type, *P* < 0.05 for fat type × time interaction and fat amount × time interaction. (B) Net iAUC of serum ghrelin, *P* < 0.001 for fixed factor fat type. HFM, high-fat meal; iAUC, incremental AUC; LFM, low-fat meal.

In response to all 4 test meals, serum ghrelin decreased in the early postprandial period (baseline until 2.0 h postprandially), followed by an increase in the later postprandial period (2.0–6.0 h postprandially). As demonstrated in Figure 1A, coconut oil–containing meals exhibited a different time-dependent pattern than canola oil–containing meals and appear to result in higher postprandial ghrelin concentrations in the later postprandial period (after 2.0 h postprandially). Additionally, higher serum ghrelin concentrations were observed after the low-fat meals than after the high-fat meals in the later postprandial period. A comparison of the net iAUC data revealed an effect of fat type, reflected by a stronger decrease of serum ghrelin after canola oil–containing meals than after coconut oil–containing meals [canola compared with coconut: –27,700 (–40,700, –14,700) min × pg/mL, *P* < 0.001]. The effect of fat amount became less relevant for the net iAUC [high fat compared with low-fat: −9500 (–22,500, 3500) min × pg/mL, *P* = 0.149] (Figure 1B).

**FIGURE 2:**
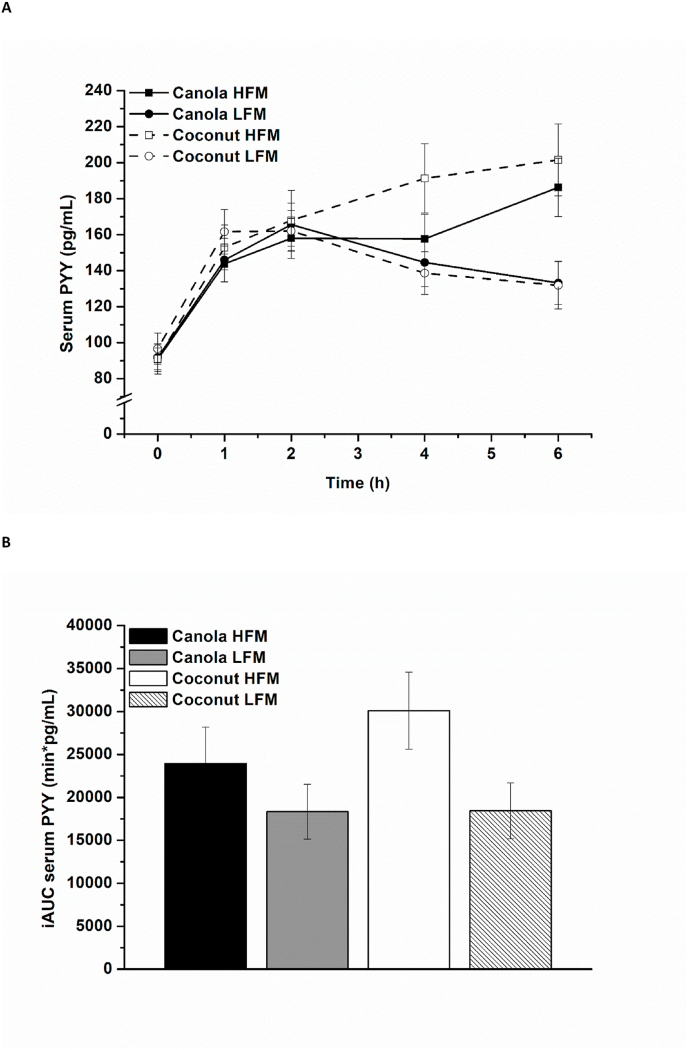
Fasting and postprandial serum PYY according to treatment condition shown as mean ± SEM (*n* = 26). (A) Serum PYY over time, *P* < 0.001 for fixed factor time and for fat amount × time interaction, *P* < 0.05 for fixed factor fat amount. (B) iAUC of serum PYY, *P* = 0.010 for fixed factor fat amount. HFM, high-fat meal; iAUC, incremental AUC; LFM, low-fat meal; PYY, Peptide YY.

Mean PYY increased in response to all 4 test meals during the first 2 h and serum concentrations were still above baseline levels at the end of the postprandial period (6.0 h). The PYY response was higher after the high-fat meals than after the low-fat meals, especially in the later postprandial period (4.0–6.0 h postprandially), regardless of fat type (Figure 2A). The iAUCs were also higher after the high-fat meals than after the low-fat meals [high-fat compared with low-fat: 8600 (2100, 15,200) min × pg/mL; fat amount effect, *P* = 0.010] (Figure 2B).

### Hunger and satiety ratings (VAS)

**FIGURE 3:**
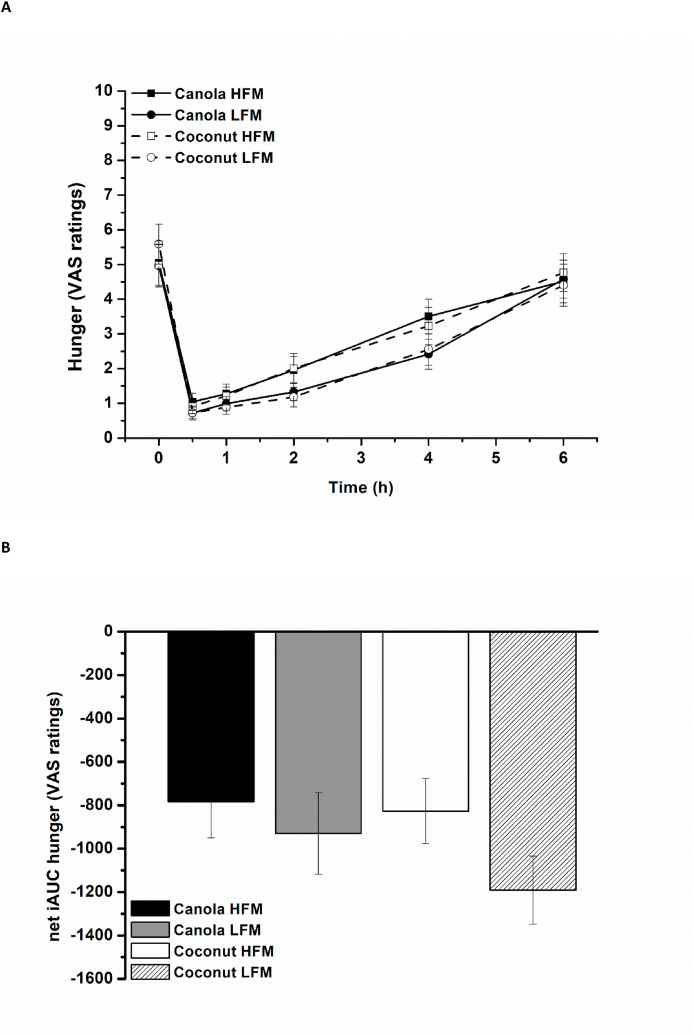
Fasting and postprandial hunger (VAS ratings) according to treatment condition shown as mean ± SEM (*n* = 29). VAS ratings range from 0 (not hungry) to 10 (hungry). (A) Hunger ratings over time, *P* < 0.001 for fixed factor time, *P* < 0.05 for fixed factor fat amount. (B) Net iAUC of hunger ratings, *P* = 0.007 for fixed factor fat amount. HFM, high-fat meal; iAUC, incremental AUC; LFM, low-fat meal; VAS, visual analog scale.

**FIGURE 4:**
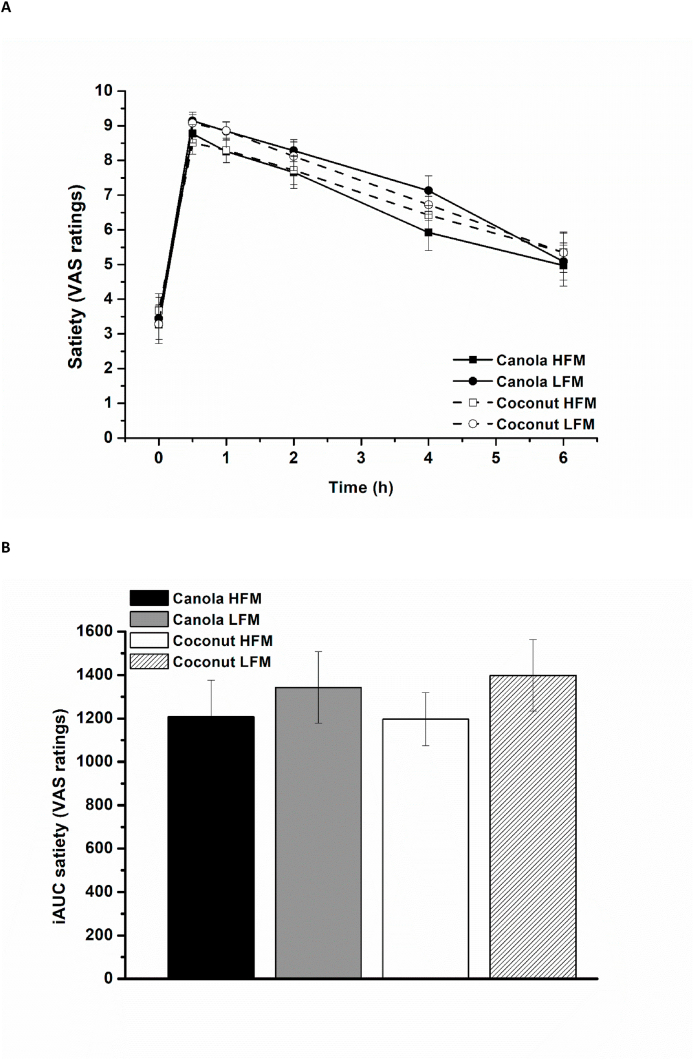
Fasting and postprandial satiety (VAS ratings) according to treatment condition shown as mean ± SEM (*n* = 29). VAS ratings range from 0 (not satiated) to 10 (satiated). (A) Satiety ratings over time, *P* < 0.001 for fixed factor time. (B) iAUC of satiety ratings. HFM, high-fat meal; iAUC, incremental AUC; LFM, low-fat meal; VAS, visual analog scale.

The feeling of hunger decreased over time in response to all 4 test meals (time effect, *P* < 0.001). The participants stated that they were less hungry after the low-fat meals than after the high-fat meals [high-fat compared with low-fat: 0.5 (0.03, 0.9), *P* = 0.039], regardless of fat type [canola compared with coconut: 0.1 (–0.3, 0.6), *P* = 0.590] (Figure 3A). The feeling of satiety increased over time (time effect, *P* < 0.001) independent of fat amount [high-fat compared with low-fat: −0.4 (–0.9, 0.03), *P* = 0.065] and fat type [canola compared with coconut: –0.02 (–0.5, 0.4), *P* = 0.943] (Figure 4A). The net iAUC data showed that participants were less hungry after the low-fat meals [high-fat compared with low-fat: 250 (71, 430), *P* = 0.007] and iAUC data revealed no relevant effect of fat amount [high-fat compared with low-fat: −160 (–400, 40), *P* = 0.108] or fat type [canola compared with coconut: 15 (–200, 250), *P* = 0.881] on satiety ratings (FIGURE 3, FIGURE 4B).

### Cognitive parameters (FAIR-2 and VVM)

**FIGURE 5:**
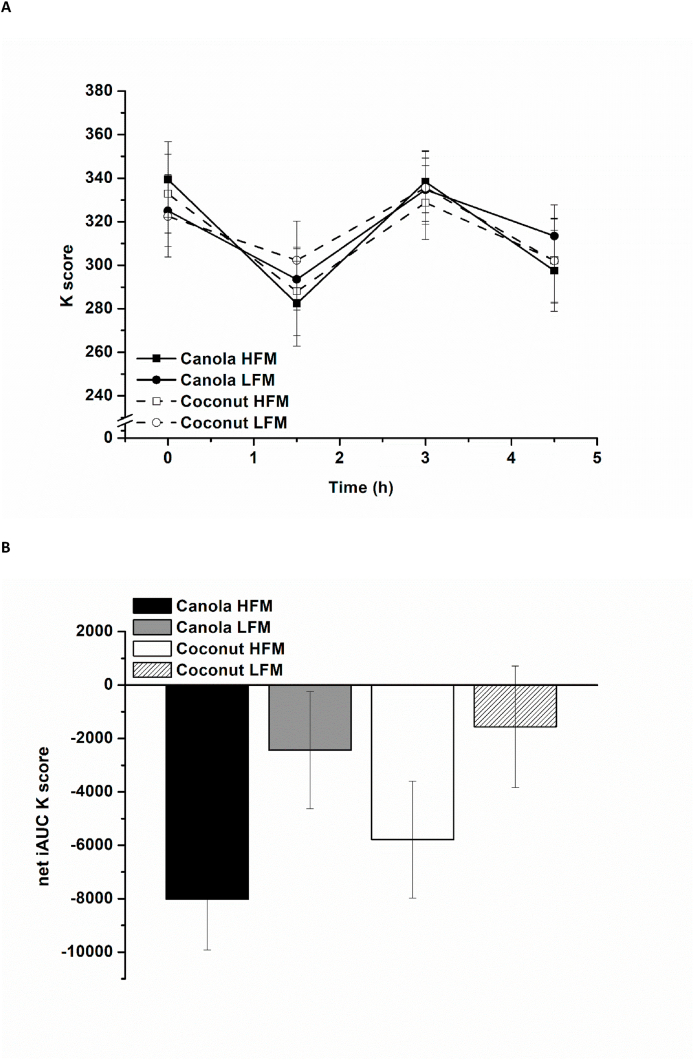
Fasting and postprandial attention (K score, FAIR-2) according to treatment condition shown as mean ± SEM (*n* = 29). The K score describes the overall test performance during FAIR-2. (A) K score over time, *P* < 0.001 for fixed factor time, *P* < 0.05 for fixed factor fat amount. (B) Net iAUC of K score, *P* = 0.003 for fixed factor fat amount. FAIR-2, Frankfurt Attention Inventory 2; HFM, high-fat meal; iAUC, incremental AUC; LFM, low-fat meal.

The K score decreased from baseline to 1.5 h postprandially, followed by an increase from 1.5 to 3.0 h postprandially and a further decrease from 3.0 to 4.5 h postprandially (time effect, *P* < 0.001) (Figure 5A). A significant fat amount effect was observed, with higher K scores after the low-fat meals compared with the high-fat meals [high-fat compared with low-fat: –12 (–25, –0.6), *P* = 0.039], regardless of fat type [canola compared with coconut: –3 (–14, 9), *P* = 0.632]. The net iAUC data showed higher K scores after the low-fat meals [high-fat compared with low-fat: –5000 (–8000; –1700), *P* = 0.003], with no fat type effect [canola compared with coconut: –1750 (−4900, 1400), *P* = 0.278] (Figure 5B).

**FIGURE 6:**
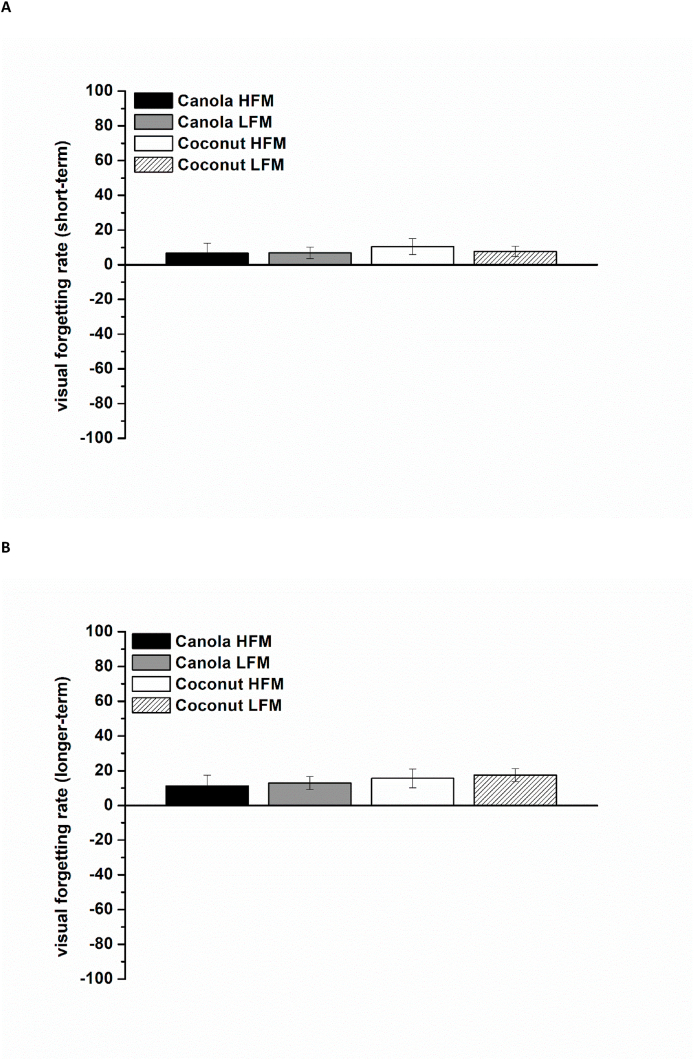
Visual forgetting rates (VVM) according to treatment condition (100 = complete forgetting of all information, –100 = complete recall of all information) shown as mean ± SEM (*n* = 29). (A) Short-term forgetting rates from 0.5 to 1.5 h postprandially. (B) Longer-term forgetting rates from 0.5 to 6.0 h postprandially. HFM, high-fat meal; LFM, low-fat meal; VVM, Visual and Verbal Memory Test.

**FIGURE 7:**
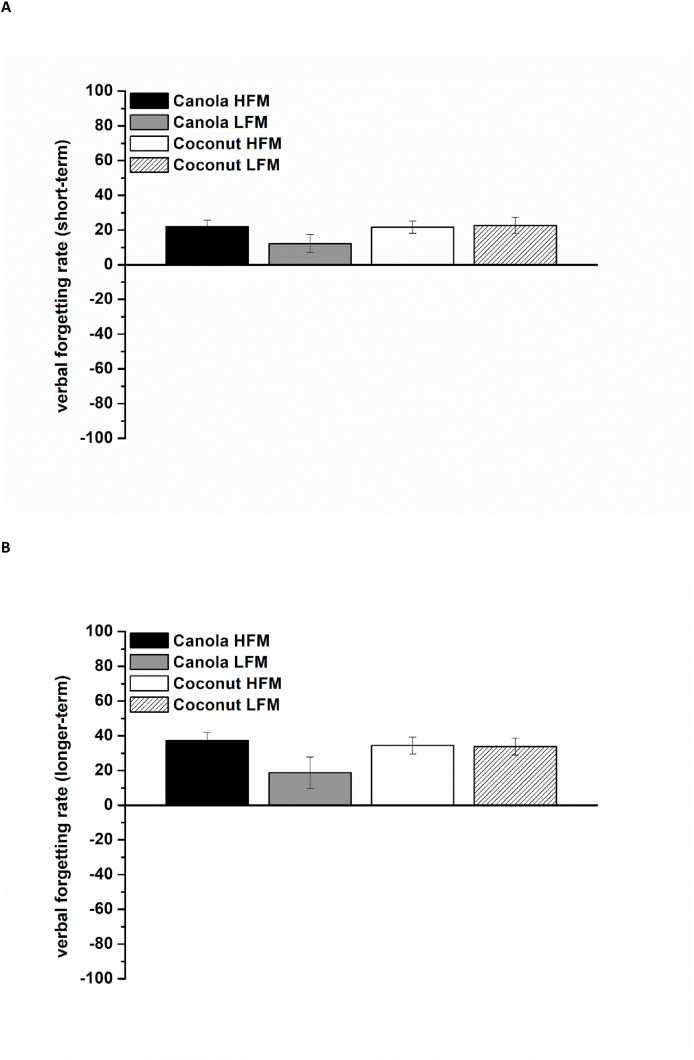
Verbal forgetting rates (VVM) according to treatment condition (100 = complete forgetting of all information, –100 = complete recall of all information) shown as mean ± SEM (*n* = 29). (A) Short-term forgetting rates from 0.5 to 1.5 h postprandially. (B) Longer-term forgetting rates from 0.5 to 6.0 h postprandially. HFM, high-fat meal; LFM, low-fat meal; VVM, Visual and Verbal Memory Test.

The short-term forgetting rates were lower compared with the longer-term forgetting rates for both the visual and verbal test form (VVM-data). Meal composition (fat amount and fat type) did not have an effect on these results (Figures 6A, B and 7A, B).

## Discussion

The aim of this exploratory analysis was to investigate the acute effects of fat amount and fatty composition (fat type) on hunger- and satiety-related parameters (specific peptide hormones and subjective ratings), attention, and memory in older adults with a risk phenotype for the development of cardiometabolic diseases for the first time. The presented data were obtained as secondary outcome measures in a trial originally designed to investigate the postprandial effects of canola and coconut oils on metabolic, inflammatory, oxidative, and endothelial parameters [20]. Our major findings were that predominantly the amount of dietary fat (50 g compared with 25 g), rather than its type (canola compared with coconut oil), might affected hunger- and satiety-related parameters, as well as postprandial attention. None of the 4 dietary interventions (“canola oil high-fat meal,” “canola oil low-fat meal,” “coconut oil high-fat meal,” “coconut oil low-fat meal”) demonstrated clear superiority in any of the assessed parameters.

All of the parameters associated with hunger (subjective ratings and serum ghrelin) decreased in the early postprandial period and then increased thereafter until the end of the observational period. In all 4 intervention groups, subjective hunger ratings and serum ghrelin concentrations were still below baseline 6.0 h postprandially. Although dietary fat is known to be a potent inhibitor of gastric emptying, suggesting that high-fat meals would be expected to reduce hunger and enhance satiety to a greater extent than low-fat meals, the participants stated that they felt less hungry after the low-fat meals than after the high-fat meals. As dietary fibers are known to improve satiety and therefore reduce hunger [29], it can be assumed that the observed fat amount effect is attributable to the higher fiber content of the low-fat meals, which could have played a role in modulating subjective hunger ratings [30]. However, it remains uncertain whether the difference in dietary fiber content between high-fat meals (6.6 g) and low-fat meals (11.2 g) was sufficient to account for the observed differences in satiety, particularly in the context of an acute intervention setting [31]. Interestingly, the fat amount effect observed for the subjective ratings of hunger was not mirrored by the serum ghrelin data; even though the fat amount effect became less relevant for the net iAUC data, the postprandial time course of ghrelin showed that higher concentrations of this appetite-stimulating hormone were observed after low-fat meals during the late postprandial period (2.0–6.0 h postprandially). This is surprising, as the postprandial decrease in ghrelin concentrations is described to be related to the energy content of the ingested meal [32], with fat typically eliciting a weaker suppression compared with, for example, carbohydrates [4,[33], [34], [35]]. Consequently, a more pronounced reduction and lower postprandial ghrelin levels would have been anticipated after consumption of the low-fat meals because of their comparatively higher carbohydrate content. However, the effect of fat amount became less relevant for the iAUC data.

Regarding the effect of fat type on postprandial serum ghrelin, the data revealed that coconut oil–containing meals resulted in higher postprandial ghrelin concentrations compared with canola oil–containing meals in the later postprandial period (2.0–6.0 h postprandially). This suggests that canola oil may suppress the feeling of hunger on a hormonal level to a greater extent than coconut oil. These results are in line with another intervention study investigating the hunger and satiety responses to high-fat meals (70% of energy) of varying fatty acid composition in women with obesity. The authors observed a more pronounced ghrelin reduction after the meals rich in PUFAs (42.3% PUFA) or MUFAs (42.4% MUFA) than after the SFA-rich meal (40.4% SFA) [7]. Just as in our study, the serum ghrelin concentration did not reflect the participants’ subjective feelings of hunger [7]. To further investigate whether the fat amount and/or fatty acid composition of the test meals influenced energy intake at a subsequent meal, the inclusion of an ad libitum meal at the end of the observational period would have been beneficial in our study. However, given the complexity of appetite regulation, which involves multiple homeostatic and hedonic mechanisms [2,36], it is likely that the inclusion of an ad libitum meal would not have led to a differential energy intake between the intervention groups.

As expected, satiety-related parameters (subjective ratings and serum PYY) showed an inverse response compared with hunger-related parameters. Both subjective satiety ratings and serum PYY increased after meal consumption and remained elevated above baseline levels in all 4 intervention groups at the end of the 6-h postprandial period. Unlike the feeling of hunger, the feeling of satiety was unaffected by fat amount. Even though iAUC data suggest a greater feeling of satiety after low-fat meals, which aligns with the observed effects for subjective ratings of hunger, these potential fat amount effects did not reach statistical significance (*P* = 0.108). The PYY response was higher after the high-fat meals than after the low-fat meals, especially in the later postprandial period (4.0–6.0 h postprandially). These results are consistent with previous findings indicating that the magnitude of the postprandial PYY increase is proportional to the energy content of a meal, with dietary fat being the most potent macronutrient in stimulating PYY release [4]. No effect of fat type on the postprandial PYY response was observed. Accordingly, the satiety-inducing potential of canola oil indicated by the serum ghrelin data was not substantiated by the observed PYY response. Overall, current evidence regarding the influence of fatty acid chain length and, in particular, the degree of saturation on PYY secretion in humans is heterogenous. Several human intervention studies suggest that differences in the duration of absorption and, consequently, the contact time of fatty acids with the PYY-secreting L-cells in the small intestine are dependent on their chain length [11,19] as well as their degree of saturation [15,19], and that longer contact times result in a greater PYY secretion. Animal studies further indicate that high-fat meals differentially affect the expression of hypothalamic orexigenic and anorexigenic neuropeptides depending on their fatty acid composition; however, this mechanism has not yet been investigated in humans [7,37]. It is plausible that, in our case, the potent effect of the total fat amount may have masked the potential effects of fatty acid composition on the PYY response.

Breakfast is often described as the most important meal of the day, as it is the first meal after an overnight fast that provides the body and brain with energy and nutrients. Furthermore, habitual breakfast consumption is associated with better cognitive performance and academic achievement [38]. In contrast to these findings, in our study the consumption of all 4 breakfast challenges led to a decrease in postprandial attention over time and K scores at 4.5 h postprandially were still below baseline for all 4 test meals. This observation is also inconsistent with the results of a previous study conducted by our research group, in which we used the FAIR-2 for the first time [39]. In this previous study, we repeatedly used the FAIR-2 version A to examine postprandial attention. This approach might have been responsible for the constant increase in the K score over time because of a “learning effect.” In our present study, we used the FAIR-2 versions A and B in an alternating order on each study day to counteract possible learning effects. Apparently, the variation in target items and test procedures at each time point (0, 1.5, 3.0, and 4.5 h) may have posed a cognitive challenge for the participants, potentially contributing to the wave-like pattern observed in the K score over the course of the observational period (decrease from baseline to 1.5 h, increase from 1.5 to 3.0 h, decrease from 3.0 to 4.5 h). Consistent with this interpretation, we observed similar K scores at time points where the same test version (A or B) was administered (0 and 3.0 h; 1.5 and 4.5 h) (Figure 5A). It remains unclear whether the repeated use of a single test version or the alternating use of the 2 different versions represents the more appropriate approach for future studies. Despite the potential influence of our approach on the time course of the K score, the data revealed that the K score was significantly higher after low-fat meals than after high-fat meals, for both the coconut and canola oil–containing meals. The reason for this fat amount effect might be the higher carbohydrate (144.1 g compared with 85.5 g) and dietary fiber (11.2 g compared with 6.6 g) content in the low-fat meals, which provoked a higher glycemic response and longer postprandial glucose availability [20]. Glucose is considered to be the predominant fuel for brain function and glucose availability and is therefore essential for general cognitive performance [[40], [41], [42]]. Interestingly, we did not find a fat amount effect on memory in our study. Instead, the participants’ memory performance decreased equally after all 4 breakfast meals, regardless of the type and amount of fat ingested. Previous human intervention studies have investigated the effects of a regular SFA, MUFA, and PUFA consumption on cognitive parameters and their results suggest that diets rich in PUFAs (especially ω-3 PUFAs) are associated with improved memory functions. On the other hand, a high intake of SFA has been linked to impaired cognitive functioning [41,43]. Overall, in studies assessing cognitive performance, a wide variety of test instruments are commonly employed. This methodological heterogeneity complicates the comparability of results across studies. Additionally, human intervention studies investigating the acute impact of the fatty acid composition of a single meal on cognitive performance are rather scarce. An intervention study investigating the acute effect of meals (∼4300 kJ/meal) enriched with SFA (100 g butter, 63% SFAs) or MUFA (80 g olive oil, 81% MUFAs) on cognitive function in lean and obese men concluded that the ingestion of SFA-enriched meals is associated with an acute decrease in cognitive function in obese participants [44]. Despite the clear differences in the fatty acid composition between coconut oil– and canola oil–containing meals (Table 2) [20], we were not able to detect any effect of fat type/fatty acid composition on memory performance or attention. Further postprandial intervention trials are warranted to clarify how meals enriched with different types of fatty acids acutely affect cognitive function, thereby contributing to a better understanding of the long-term effects of dietary fat on cognitive outcomes.

The main strengths of the postprandial trial on which the presented data are based include its controlled crossover design and the high rate of treatment compliance. Furthermore, the trial focused on regular and realistically designed test meals and not on the administration of nutrient solutions (e.g., fat tolerance tests). Even though the results are of limited transferability to other populations (e.g., metabolically healthy individuals), the study was conducted in a clinically relevant cohort characterized by an increased risk of cardiometabolic diseases.

One limitation of the present work is its exploratory nature and the fact that no a priori sample size calculation was performed for the presented outcome measures. However, the precision of the estimates for the differences were high enough to allow for the identification of clear trends. In our opinion, our results offer valuable input for planning future intervention studies (e.g., sample size calculations) that aim to investigate the effects of fat amount and fatty acid composition on hunger, satiety, and cognition. Another potential limitation concerns the alternating use of the FAIR-2 versions A and B to assess postprandial attention. This approach may have presented a cognitive challenge—manifesting as potential confusion—for the participants, which could have influenced the K score pattern and introduced interpretative bias. It remains unclear whether the repeated use of a single test version, as applied in our previous study [39], or the alternating use of the 2 different versions, as applied in this study, represents the more appropriate approach for future studies. Importantly, any potential “learning effects” or participants’ confusion resulting from either approach appears to only affect the time line of the K score and does not confound or bias the detection of treatment effects. This is also supported by our findings of significant effects of fat amount/carbohydrate content on postprandial attention.

In conclusion, our data suggest that in older adults with a risk phenotype for cardiometabolic diseases, predominantly the amount of dietary fat, rather than its type (canola compared with coconut oil), affects hunger- and satiety-related parameters, as well as postprandial attention. However, fat type influenced the postprandial ghrelin response, with lower concentrations observed following canola oil–containing meals. This indicates that canola oil suppresses postprandial hunger at the hormonal level to a greater extent than coconut oil.

## Author contributions

The authors’ responsibilities were as follows – CD, HFK, MW, RN, SE: designed the study; CD, TBS, HFK, CW, CK, MC, SE: conducted the study; BS-W, CK: analyzed blood samples; CD, RN: performed statistical analysis; CD: wrote the first draft of the manuscript, which was finalized in close collaboration with SE; and all authors: declare responsibility for the final content and have read and approved the final manuscript.

## Data availability

The data will be made available upon request.

## Funding

This study was financially supported by the Union for the Promotion of Oil and Protein Crops e. V. (UFOP), without involvement or restrictions regarding the study design; collection, analysis, and interpretation of data; writing of the report; or submission of the report for publication.

## Conflict of interest

The authors declare no conflicts of interest.
